# A New Risk Model Based on 7 Quercetin-Related Target Genes for Predicting the Prognosis of Patients With Lung Adenocarcinoma

**DOI:** 10.3389/fgene.2022.890079

**Published:** 2022-05-13

**Authors:** Yun-Qiang Zhang, Kai Li, Qiang Guo, Dan Li

**Affiliations:** ^1^ Department of Thoracic Surgery, Beilun District People’s Hospital, Ningbo, China; ^2^ Department of Hepatobiliary and Pancreatic Surgery, The People’s Hospital of jianyang City, Jianyang, China; ^3^ Department of Thoracic Surgery, Huanggang Central Hospital, Huanggang, China; ^4^ Department of Oncology, Huanggang Central Hospital, Huanggang, China

**Keywords:** LUAD, risk model, quercetin, prognosis, GSEA

## Abstract

**Background:** Studies have reported that quercetin inhibits the growth and migration of lung adenocarcinoma (LUAD). This study aimed to explore the roles and mechanisms of quercetin target genes in the progression of LUAD.

**Methods:** The quercetin structure and potential target genes of quercetin were explored in the Traditional Chinese Medicine Systems Pharmacology and SwissTargetPrediction databases. The differentially expressed quercetin target genes were identified in The Cancer Genome Atlas (TCGA) database, and the clinical values of quercetin target genes were explored. Subsequently, a risk model was constructed *via* the Cox regression and survival analysis to evaluate the potential effects and possible mechanisms of quercetin target genes.

**Results:** The quercetin differential target genes involved in biological processes such as the oxidation-reduction process, cell proliferation, G2/M transition of the mitotic cell cycle, and were related to the lung cancer. NEK2, TOP2A, PLK1, CA4, CDK5R1, AURKB, and F2 were related to the prognosis, and were independent factors influencing the prognosis of LUAD patients. The risk model was related to the gender, clinical stage, T stage, lymph node metastasis, and survival status of LUAD patients, and was independent risk factor associated with poor prognosis. In the high-risk group, the risk model involved signaling pathways such as cell cycle, DNA replication, spliceosome, and homologous recombination.

**Conclusion:** The quercetin potential target genes NEK2, TOP2A, PLK1, CA4, CDK5R1, AURKB, and F2 were related to the diagnosis and prognosis of LUAD patients. A risk model based on 7 quercetin target genes could be used to assess the prognosis of patients with LUAD.

## Introduction

*Houttuynia cordata* is the herbal medicine included in the Chinese pharmacopeia and is derived from the dry aerial part of the saururus chinensis plant family. At present, *H. cordata* has shown anti-tumor progression effects in cancer (Kim et al., 2017; Lou et al., 2019; Subhawa et al., 2020; Liu et al., 2021). For example, *H. cordata* enhances HIF-1A/FOXO3 signaling transduction, causing the up-regulation of MEF2A expression in HepG2 cells, inhibiting the expression of Bcl-2 family proteins (Bax, Bcl-2 and Bcl-Xl), promoting cell apoptosis, and inhibiting the growth of liver cancer cells transplanted into nude mice (Kim et al., 2017). *H. cordata* and 2-undecanone can significantly inhibit benzo(a)pyrene from inducing lung tumors without causing significant systemic toxicity in mice (Lou et al., 2019). In addition, *H. cordata* component quercetin features important biological values in the progression of lung cancer (Lee et al., 2015; Sonoki et al., 2015; Chuang et al., 2016; Klimaszewska-Wiśniewska et al., 2017; Dong et al., 2020). For example, quercetin reduces the viability of lung cancer cells, inhibits the expression of HSP70, and increases gemcitabine-induced cancer cell death. The combination therapy of quercetin and gemcitabine significantly down-regulates the expression of HSP70 (Lee et al., 2015). It has been reported that quercetin inhibits the proliferation, migration and invasion of non-small cell lung cancer (NSCLC) cells *in vitro*, and inhibits the growth of solid tumors *in vivo*. The Src expression and Fn14/NF-kappaB (NF-kB) signaling pathway are inhibited by the quercetin. Src overexpression in NSCLC cells leads to NSCLC cell proliferation and metastasis, and activating the Fn14/NF-kB signaling pathway inhibits the anti-NSCLC effect of quercetin (Dong et al., 2020). Therefore, the quercetin has the anti-tumor roles in lung cancer.

It has been confirmed that the quercetin has the anti-tumor properties in lung cancer and lung adenocarcinoma (LUAD) (Lee et al., 2015; Dong et al., 2020; Chuang et al., 2016; Klimaszewska-Wiśniewska et al., 2017; Sonoki et al., 2015). However, the mechanisms of quercetin in the development of LUAD has not yet been fully revealed. The Cancer Genome Atlas (TCGA) (https://portal.gdc.cancer.gov/) database is a cancer database from the United States with reliable gene expression data and complete clinical information from a large sample of cancer patients (Guo Q et al., 2020; Liu et al., 2020). This study aimed to screen the hub target genes of quercetin in the progression of LUAD using the large data of TCGA database to elucidate the biological functions and mechanisms involved. A risk model of quercetin could then be constructed to assess the potential application as a prognostic tool for LUAD patients (Supplementary Figure S1), and evaluate the hub target genes as new therapeutic targets.

## Materials and Methods

### Screening of Quercetin Target Genes

The quercetin structure was obtained from the Traditional Chinese Medicine Systems Pharmacology (TCMSP) Database (http://sm.nwsuaf.edu.cn/lsp/tcmsp.php), and the possible target genes of quercetin were predicted *via* the SwissTargetPrediction (http://www.swisstargetprediction.ch) database. Moreover, the quercetin structure from the pubchem (https://pubchem.ncbi.nlm.nih.gov/) database, and protein receptor structures from the PDB (http://www.rcsb.org/) database were downloaded to visualize molecular docking.

### Identification of Quercetin Target Genes

The transcriptome data of gene expression and 522 clinical data of LUAD were downloaded from the TCGA database (Chen et al., 2019; Guo Q et al., 2020). The gene expression data included the 59 normal lung tissues and 535 LUAD tissues. The expression levels of quercetin target genes were identified *via* the Limma package, and the screening criteria was the logFC > 1 or < −1 and *p* < 0.05. The correlation between differentially expressed genes (DEGs) were analyzed using the correlation analysis. The roles of DEGs in LUAD were analyzed *via* the receiver operating characteristic (ROC) analysis. The area under the curve (AUC) and the *p* value were used as evaluation indicators.

### Bioinformatics Analysis

The biological process (BP), cellular component (CC), molecular function (MF), signaling pathways and diseases of the quercetin related DEGs were explored in the DAVID database. We constructed a protein-protein interaction (PPI) network of the target genes of quercetin using the STRING (version: 11.5) database, and Cytoscape (version: 3.8.2) software was used for visualization.

### Cox Regression Analysis

Univariate Cox regression analysis was used to analyze whether the DEGs of quercetin affected the prognosis of patients with LUAD, and the screening criteria was the *p* < 0.05. The independent factors affecting the prognosis of LUAD patients were screened using multivariate Cox regression analysis and akaike information standard (AIC), and the LUAD patients were divided into the high- and low-risk groups based on gene expression values. Subsequently, the clinical data were sorted out, and univariate Cox regression analysis was used to analyze the influence of clinicopathological characteristics of the high- and low-risk groups on the prognosis of LUAD patients.

### Clinical Prognostic Value Analysis

The mortality of LUAD patients in the high- and low-risk groups was assessed by the Kaplan-Meier (K-M) survival analysis. The relationship between high- and low-risk and clinicopathological characteristics (age, gender, clinical stage, T stage, N stage, and M stage) of LUAD patients was evaluated *via* the correlation analysis. Furthermore, the roles of risk model related DEGs in the prognosis of LUAD patients were identified in the K-M plotter database and Gene Expression Profiling Interactive Analysis (GEPIA) database. *p* < 0.05 was considered statistically significant.

### Consensus Clustering

The NEK2, TOP2A, PLK1, CA4, CDK5R1, AURKB, and F2 expression data of 535 LUAD tissues were analyzed using the Consensus ClusterPlus package of R (version: 4.1.1), and principal component analysis (PCA) was carried out in cluster1 and cluster2 groups.

### The Signaling Mechanism Involved in the Risk Model

The gene expression profiles of LUAD patients were divided into the high and low expression groups according to the median riskscore vlaue. The gene set enrichment analysis [GSEA (version: 4.1.0)] was used to assess the impact of potential mechanisms in the constructed risk model on the progression of LUAD. Each analysis performed 1,000 permutations of the genome. Gene sets with NOM *p* < 0.05 were considered significantly enriched.

### Statistical Analysis

The data obtained from the TCGA database were processed using the Perl and R. The prognosis-related DEGs in LUAD were assessed using survival analysis, Cox regression analysis. Univariate Cox regression, multivariate Cox regression, and AIC method were used to construct the risk model for LUAD patients. *p* < 0.05 was considered the statistically significant.

## Results

### Screening and Identification of Quercetin Differentially Expressed Target Genes

**FIGURE 1:**
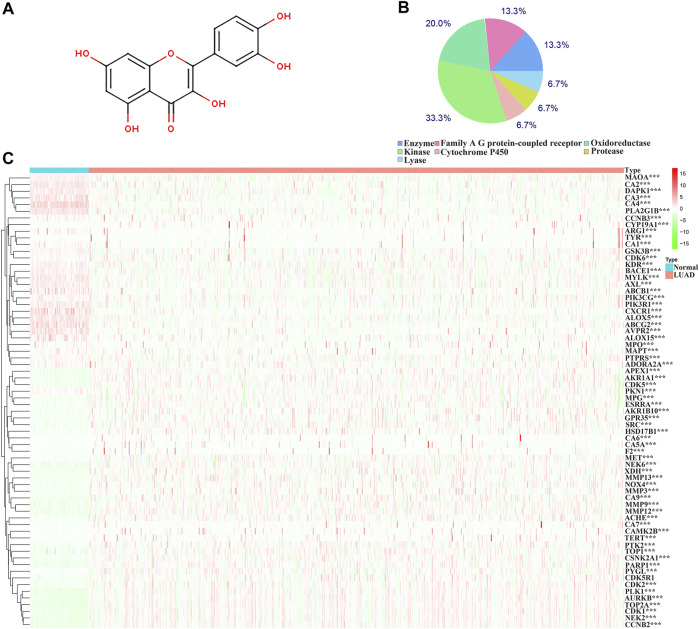
Structure and target genes of quercetin. **(A)** Structure; **(B)** The distribution ratio of quercetin target genes; **(C)** Quercetin-related differentially expressed genes in TCGA LUAD tissues. Note: TCGA, The Cancer Genome Atlas; LUAD, lung adenocarcinoma.

The structure of quercetin was obtained from the TCMSP database (Figure 1A). Figure 1B shows the distribution of quercetin target genes in the SwissTargetPrediction database (Supplementary Table S1). In the TCGA database, compared with normal lung tissues, there were the 79 DEGs in LUAD tissues (Figure 1C; Supplementary Table S2). Among them, 40 DEGs exhibited a fold change greater than 1 (Supplementary Figure S2; Table 1).

**TABLE 1:** Quercetin differentially expressed target genes in LUAD tissues.

Gene	Normal	LUAD	logFC	*p* value
ALOX5	46.25523982	14.52732521	−1.670847708	5.93E-29
CAMK2B	0.049236775	0.421586942	3.098022011	0.000194154
MYLK	10.57165139	5.023807105	−1.073347781	8.14E-21
TYR	0.000583999	0.00931391	3.995349033	8.77E-05
ABCB1	1.908949012	0.719293508	−1.40812608	2.14E-14
HSD17B2	0.222337618	0.7371236	1.729154488	0.001096623
NOX4	0.344120878	0.952886641	1.469389171	2.16E-12
ACHE	0.852870206	5.110213705	2.582985518	3.06E-08
MMP9	8.445666361	37.58516834	2.153880305	7.65E-16
MET	17.25153837	46.31649801	1.424801159	0.000129599
CA9	0.269006771	13.52854628	5.652220522	1.57E-25
NEK2	0.372781618	5.481985943	3.878296003	1.13E-33
ABCG2	3.891805777	1.047588969	−1.89336694	5.70E-30
AVPR2	1.234203422	0.358546929	−1.783346333	1.39E-27
CA6	0.011849034	0.281894217	4.572312488	0.000492085
TOP2A	1.273108254	20.86271818	4.034500134	4.34E-35
MMP13	0.172913296	12.74525631	6.203767768	4.26E-26
CYP19A1	0.033011161	0.096082436	1.541318843	6.80E-08
PARP1	13.51054369	28.90555188	1.097260884	5.56E-28
MMP3	0.074374208	1.225321	4.042213541	3.35E-18
CCNB2	0.85272533	7.724280418	3.179247523	3.47E-33
XDH	0.236332784	4.310917397	4.18910324	3.48E-28
ALOX15	10.25568279	2.588697597	−1.986125195	1.07E-12
CXCR1	2.556918658	0.390156367	−2.712281917	1.80E-23
TERT	0.00486791	0.25059112	5.68588908	4.03E-31
CA3	5.164310546	1.361228429	−1.92366657	2.46E-22
PLK1	0.577680656	5.911287315	3.355128254	2.65E-34
CA4	18.53023779	1.012544663	−4.193823944	3.12E-34
CA7	0.011111517	0.084740592	2.930997398	4.25E-13
CDK1	1.559918253	9.505668682	2.60731769	3.27E-29
PLA2G1B	28.89106922	6.982282146	−2.048853037	2.94E-28
CA5A	0.008305728	0.038347433	2.206951378	1.22E-10
CDK5R1	0.49372022	1.592183329	1.689240827	6.69E-21
GPR35	0.34123397	2.031166595	2.573475395	6.85E-15
AURKB	0.562161788	6.770060245	3.590111376	8.17E-33
F2	0.004497974	0.803636905	7.481124576	3.09E-09
AKR1C1	3.523312266	34.33050626	3.284486883	0.018253344
AKR1B10	0.273728493	50.59486695	7.530101598	3.24E-14
AKR1C4	0.0328767	1.159276664	5.140015589	0.006154793
MMP12	1.174265463	20.82348939	4.148381345	4.56E-23

Note: DEGs, differentially expressed genes; LUAD, lung adenocarcinoma.

### The Biological Functions Involved in the Differential Expression of Quercetin Target Genes

**FIGURE 2:**
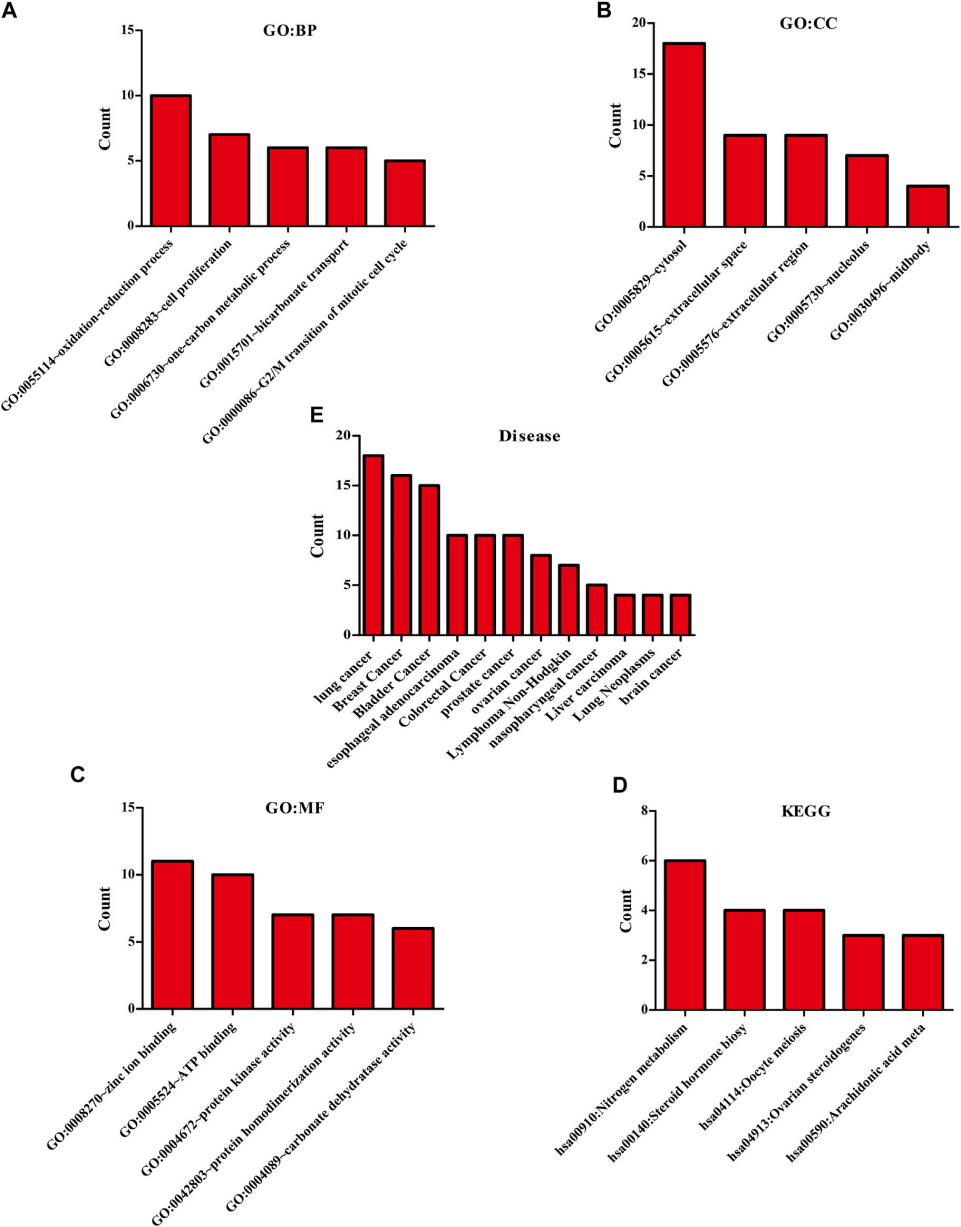
The biological functions, mechanisms and diseases of 40 quercetin-related differentially expressed target genes were explored in the DAVID database.

The biological functions involved in 40 DEGs were explored using gene ontology (GO) in the DAVID database. The GO (BP) results showed that the DEGs were involved in the oxidation-reduction process, cell proliferation, G2/M transition of the mitotic cell cycle, positive regulation of reactive oxygen species metabolic process, mitotic nuclear division, and others. The GO (CC) results indicated that the DEGs were involved in the cytosol, spindle microtubule, proteinaceous extracellular matrix, and others. The GO (MF) results showed that the DEGs were involved in protein kinase activity, cyclin-dependent protein serine/threonine kinase activity, protein serine/threonine kinase activity, oxidoreductase activity, and others (Figures 2A–C; Table 2). Furthermore, KEGG results demonstrated that the DEGs were involved in nitrogen metabolism, steroid hormone biosynthesis, oocyte meiosis, ovarian steroidogenesis and arachidonic acid metabolism signaling mechanisms (Figure 2D). In addition, the DEGs of quercetin were associated with lung cancer, breast cancer, chronic obstructive pulmonary disease, bladder cancer, esophageal adenocarcinoma, liver carcinoma, colorectal cancer, and other cancers (Figure 2E).

**TABLE 2:** The biological functions of 40 differentially expressed target genes were explored in the DAVID database.

Type	Term	Count	*p* value
BP	one-carbon metabolic process	6	7.07E-09
BP	bicarbonate transport	6	5.26E-08
BP	oxidation-reduction process	10	6.51E-06
BP	daunorubicin metabolic process	3	1.46E-04
BP	doxorubicin metabolic process	3	1.46E-04
BP	cell proliferation	7	1.83E-04
BP	G2/M transition of mitotic cell cycle	5	2.79E-04
BP	collagen catabolic process	4	4.38E-04
BP	extracellular matrix disassembly	4	7.24E-04
BP	positive regulation of protein localization to nucleus	3	0.001073426
BP	positive regulation of reactive oxygen species metabolic process	3	0.002194392
BP	mitotic nuclear division	5	0.002545347
BP	mitotic nuclear envelope disassembly	3	0.004675418
BP	retinoid metabolic process	3	0.008822724
BP	cellular response to jasmonic acid stimulus	2	0.009258647
BP	anaphase-promoting complex-dependent catabolic process	3	0.014469739
BP	xenobiotic transport	2	0.016147767
CC	Cytosol	18	2.15E-04
CC	midbody	4	0.002628974
CC	spindle microtubule	3	0.003988715
CC	extracellular space	9	0.006820729
CC	nucleolus	7	0.009188997
CC	kinetochore	3	0.012998427
CC	extracellular region	9	0.01886576
CC	proteinaceous extracellular matrix	4	0.019
MF	carbonate dehydratase activity	6	9.94E-11
MF	protein kinase activity	7	1.60E-04
MF	zinc ion binding	11	2.43E-04
MF	aldo-keto reductase (NADP) activity	3	5.36E-04
MF	electron carrier activity	4	0.001165639
MF	metalloendopeptidase activity	4	0.002239619
MF	serine-type endopeptidase activity	5	0.002761336
MF	cyclin-dependent protein serine/threonine kinase activity	3	0.002784583
MF	17-alpha,20-alpha-dihydroxypregn-4-en-3-one dehydrogenase activity	2	0.004615378
MF	iron ion binding	4	0.005253912
MF	ATP binding	10	0.006038782
MF	protein homodimerization activity	7	0.006175012
MF	endopeptidase activity	3	0.006899351
MF	indanol dehydrogenase activity	2	0.006915278
MF	collagen binding	3	0.008459805
MF	protein serine/threonine kinase activity	5	0.010776455
MF	oxidoreductase activity	4	0.010944738
MF	xenobiotic-transporting ATPase activity	2	0.011499553
MF	retinal dehydrogenase activity	2	0.016063207
MF	bile acid binding	2	0.018337329
MF	oxidoreductase activity, acting on NAD(P)H, quinone or similar compound as acceptor	2	0.018337329

BP, biological process; CC, cellular component; MF, molecular functions.

### Correlation Analysis of the Differentially Expressed Target Genes

Correlation analysis revealed a partial correlation between the DEGs expression levels in 535 LUAD tissues (Supplementary Figure S3). The degree of color was positively correlated with the degree of correlation. Furthermore, the PPI network constructed from the String database revealed the protein interaction relationship between quercetin differentially expressed target genes (Supplementary Figure S4).

### Evaluating the Diagnostic Value of Quercetin Differentially Expressed Target Genes in Lung Adenocarcinoma

**FIGURE 3:**
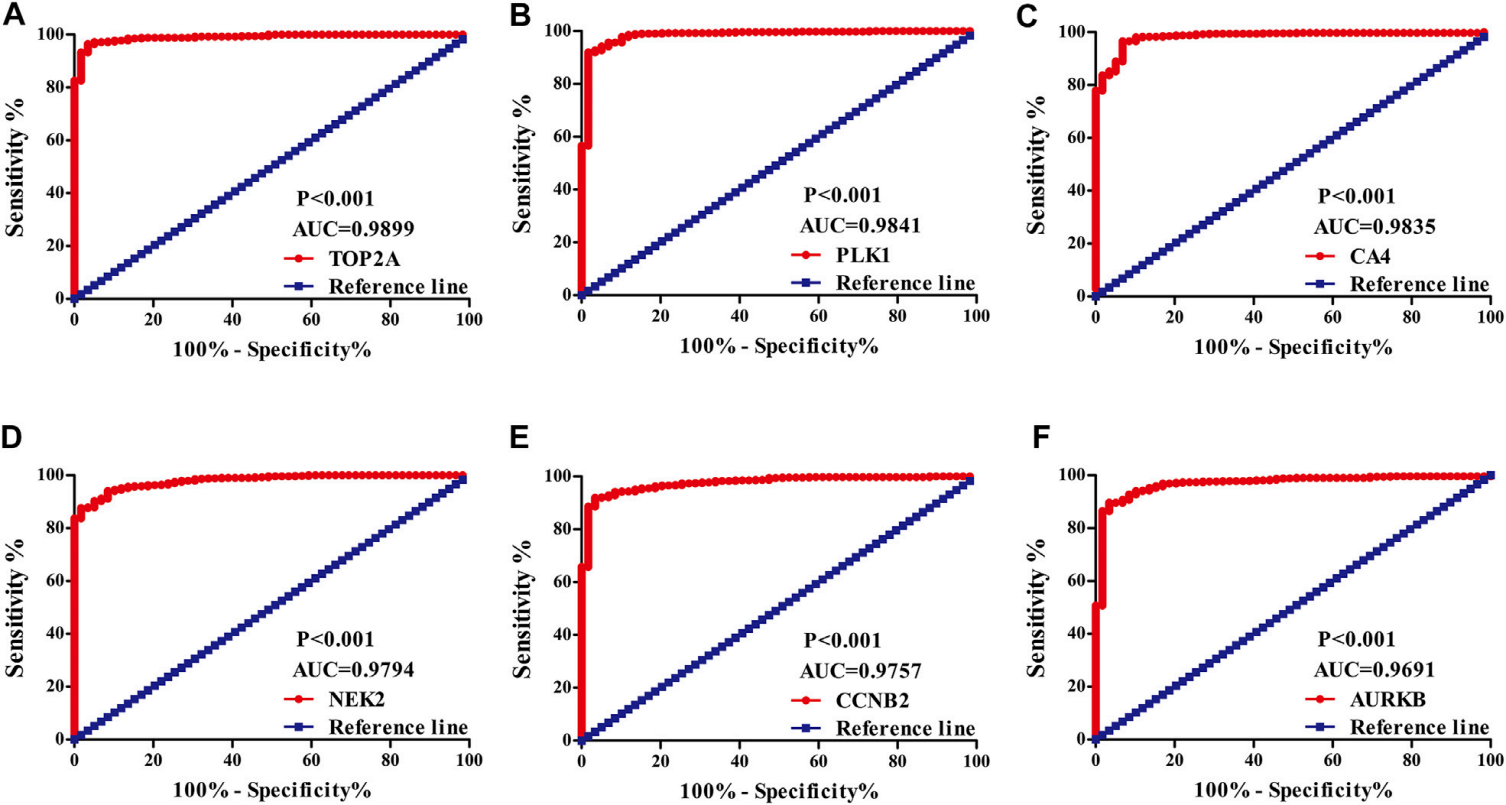
The diagnostic value of quercetin-related differentially expressed target genes in LUAD *via* the ROC analysis. Note: ROC, Receiver operating characteristic; LUAD, lung adenocarcinoma.

The diagnostic values of the DEGs in LUAD were evaluated *via* the ROC analysis. The results shows that the AUC of ALOX5 was 0.9426, CAMK2B was 0.6477, MYLK was 0.8709, ABCB1 was 0.8209, HSD17B2 was 0.6294, NOX4 was 0.7748, ACHE was 0.7195, MMP9 was 0.8195, MET was 0.6517, CA9 was 0.9139, NEK2 was 0.9794, ABCG2 was 0.9508, AVPR2 was 0.9313, CA6 was 0.6369, TOP2A was 0.9899, MMP13 was 0.9188, CYP19A1 was 0.7139, PARP1 was 0.9346, MMP3 was 0.8448, CCNB2 was 0.9757, XDH was 0.9363, ALOX15 was 0.7823, CXCR1 was 0.8957, TERT was 0.9597, CA3 was 0.8853, PLK1 was 0.9841, CA4 was 0.9835, CA7 was 0.7873, CDK1 was 0.9593, PLA2G1B was 0.9373, CA5A was 0.7564, CDK5R1 was 0.8686, GPR35 was 0.806, AURKB was 0.9691, F2 was 0.7314, AKR1C1 was 0.5859, AKR1B10 was 0.7992, AKR1C4 was 0.6086, and MMP12 was 0.8884 (Figure 3).

### Screening and Constructing a Risk Model for Lung Adenocarcinoma Patients

**FIGURE 4:**
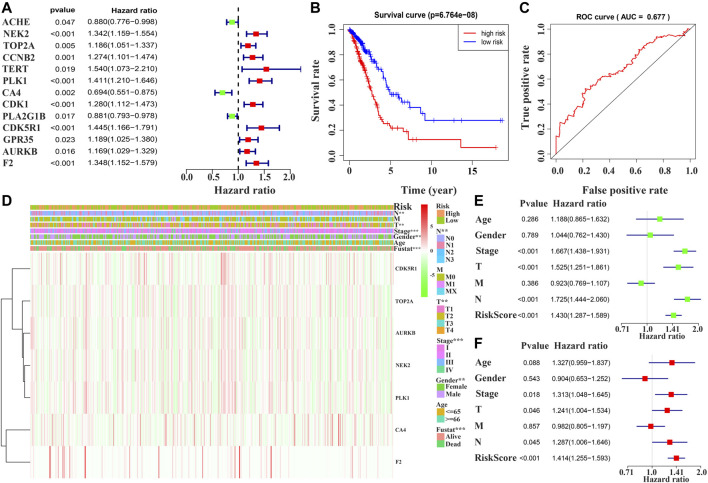
Prognostic value of quercetin-related differentially expressed target genes in patients with LUAD *via* the COX and survival analysis. Note: LUAD, lung adenocarcinoma.

**FIGURE 5:**
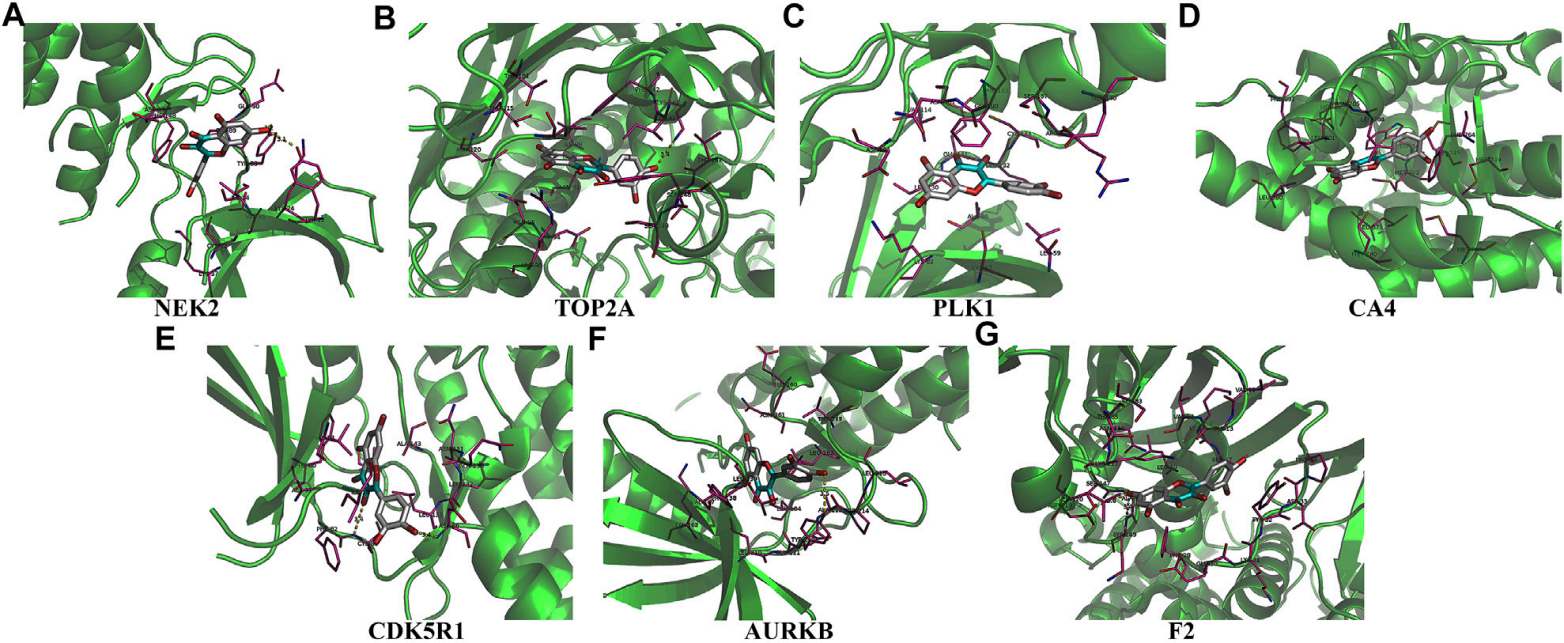
The molecular docking between quercetin and risk model genes. **(A)** NEK2; **(B)** TOP2A; **(C)** PLK1; **(D)** CA4; **(E)** CDK5R1; **(F)** AURKB; **(G)** F2.

Univariate Cox regression analysis demonstrated that ACHE, NEK2, TOP2A, CCNB2, TERT, PLK1, CA4, CDK1, PLA2G1B, CDK5R1, GPR35, AURKB, and F2 were independent factors influencing the prognosis of LUAD patients (Figure 4A; Table 3). On this basis, multivariate Cox regression analysis revealed that NEK2, TOP2A, PLK1, CA4, CDK5R1, AURKB, and F2 were independent factors affecting the prognosis of LUAD patients (Table 4), which were used to construct a risk model. Furthermore, K-M survival analysis confirmed that the prognosis of LUAD patients in the high-risk group was worse (Figure 4B), with the AUC being 0.677 (Figure 4C). The risk factors were further evaluated by correlation analysis and were found to be related to the gender, clinical stage, T stage, lymph node metastasis, and survival status of LUAD patients (Figure 4D). Univariate and multivariate Cox regression analysis showed that the high riskscore was a risk factor for poor prognosis in LUAD patients (Figures 4E,F; Table 5). Quercetin structure and risk factor structures were visualized *via* molecular docking (Figure 5).

**TABLE 3:** Univariate Cox regression analysis showed the prognostic factors of LUAD patients.

Gene	HR	HR.95L	HR.95H	*p* value
ACHE	0.879992393	0.77566432	0.998352755	0.047081185
NEK2	1.341665075	1.158628429	1.5536173	8.59E-05
TOP2A	1.18555718	1.051427252	1.33679798	0.005459325
CCNB2	1.274097559	1.101460121	1.473793339	0.001110962
TERT	1.540215074	1.073184817	2.210488294	0.019122856
PLK1	1.411101677	1.209796055	1.645903816	1.16E-05
CA4	0.694066492	0.550670476	0.8748032	0.001983239
CDK1	1.279842754	1.112209482	1.472741873	0.000571705
PLA2G1B	0.880534921	0.793089432	0.97762209	0.017122649
CDK5R1	1.445138252	1.166379866	1.790518362	0.000758367
GPR35	1.189343125	1.024742661	1.380382726	0.022515462
AURKB	1.169287666	1.029066755	1.328615116	0.016413919
F2	1.348451789	1.151870264	1.578582487	0.000200306

LUAD, lung adenocarcinoma.

**TABLE 4:** Risk model factors of LUAD patients were shown *via* the multivariate Cox regression analysis.

Gene	Coef	HR	HR.95L	HR.95H	*p* value
NEK2	0.38649321	1.471810403	1.018030893	2.127858671	0.039880939
TOP2A	−0.29936995	0.741285121	0.557509892	0.985639248	0.039448952
PLK1	0.318594941	1.375194177	0.984673167	1.920595674	0.061575757
CA4	−0.330770782	0.718369814	0.562858176	0.916847638	0.007873829
CDK5R1	0.317598825	1.373825006	1.067818786	1.767523828	0.013497859
AURKB	−0.232159508	0.792819652	0.607344339	1.034936791	0.08774605
F2	0.25208487	1.286705236	1.084252134	1.526960668	0.003900981

LUAD, lung adenocarcinoma.

**TABLE 5:** Prognosis-related clinicopathological features of LUAD patients were shown *via* the multivariate Cox regression analysis.

Factor	HR	HR.95L	HR.95H	*p* value
Age	1.326933683	0.958750713	1.836507629	0.088022326
Gender	0.903890066	0.652674012	1.251799881	0.543052004
Stage	1.31286743	1.047634521	1.645250185	0.018073597
T stage	1.240751892	1.003591762	1.533955653	0.046251584
M stage	0.981885602	0.805186207	1.19736196	0.856690249
N stage	1.286736226	1.006025223	1.64577396	0.044664006
Riskscore	1.413904145	1.254549916	1.593499713	1.37E-08

LUAD, lung adenocarcinoma.

### The Value of Identifying Model Factors in the Prognosis of Lung Adenocarcinoma Patients

**FIGURE 6:**
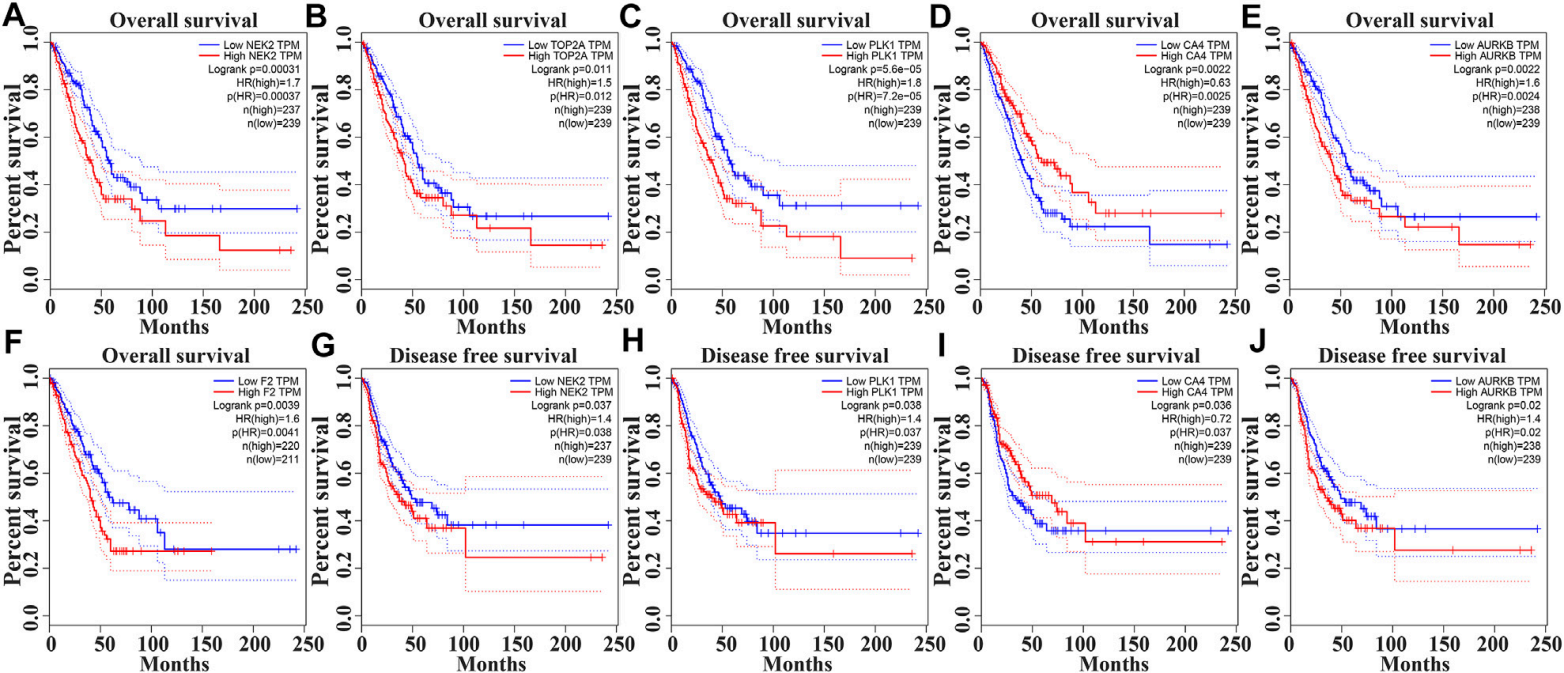
The risk model factors were correlated with the prognosis of LUAD patients in the GEPIA database. Note: LUAD, lung adenocarcinoma; GEPIA, gene expression profiling interactive analysis.

**FIGURE 7:**
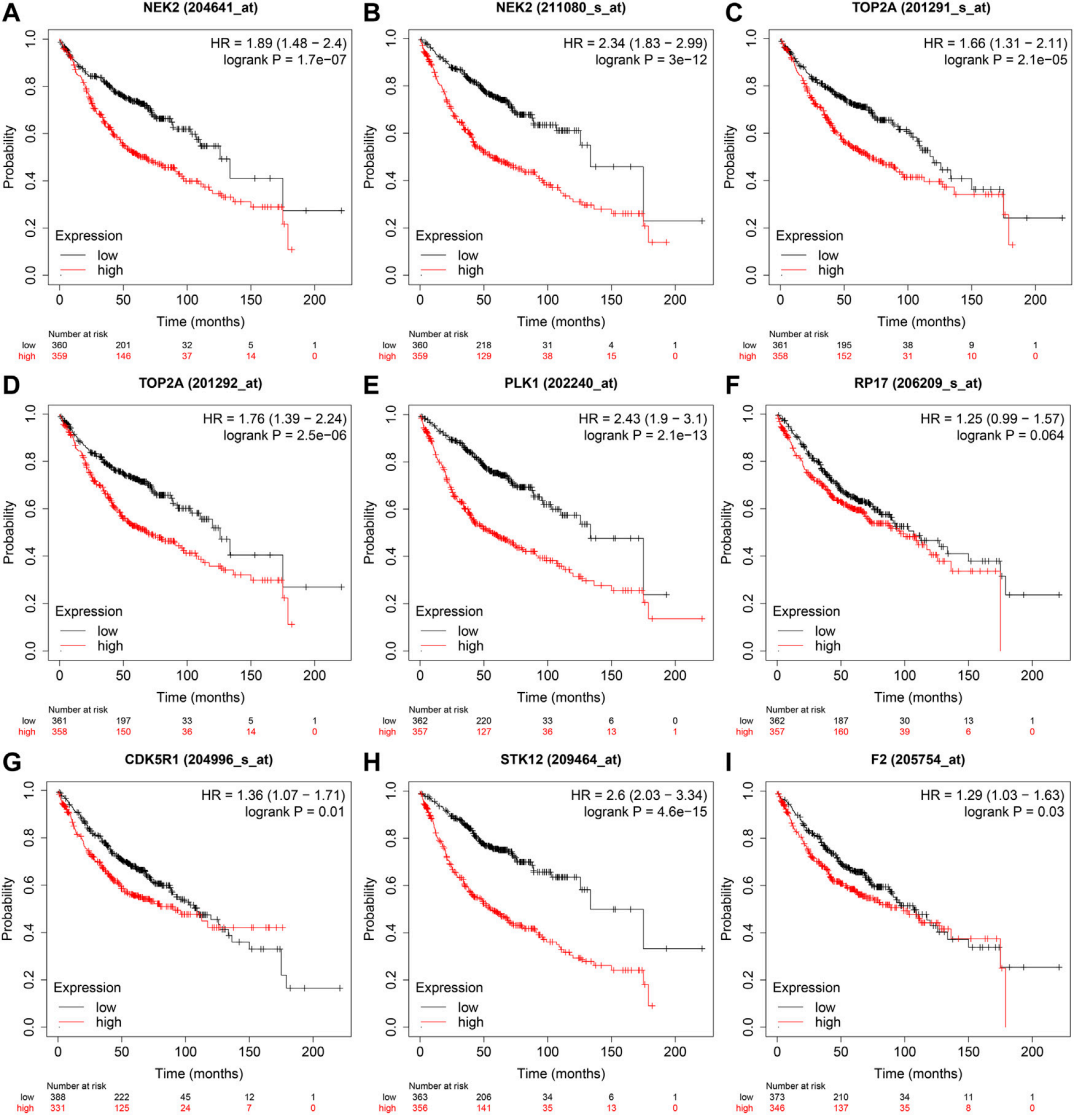
The risk model factors were correlated with the OS of LUAD patients in the Kaplan-Meier Plotter database. Note: OS, overall survival; LUAD, lung adenocarcinoma.

In the GEPIA database, the expression levels of NEK2, TOP2A, PLK1, AURKB, F2, and CA4 were confirmed to be related to the OS of LUAD patients (Figures 6A–F). Moreover, the expression levels of NEK2, PLK1, CA4, and AURKB were correlated with the disease-free survival (DFS) of LUAD patients (Figures 6G–J). In the K-M Plotter database, the expression levels of NEK2, TOP2A, PLK1, RP17, CDK5R1, STK12, and F2 were correlated with the OS in LUAD patients (Figures 7A–I). Furthermore, the expression levels of NEK2, TOP2A, PLK1, RP17, CDK5R1, and STK12 were correlated with the progression-free survival (PFS) in LUAD patients (Supplementary Figures S5A–I).

### Consensus Clustering of the Prognostic Factors Identified two Clusters of Lung Adenocarcinoma With Different Outcomes

**FIGURE 8:**
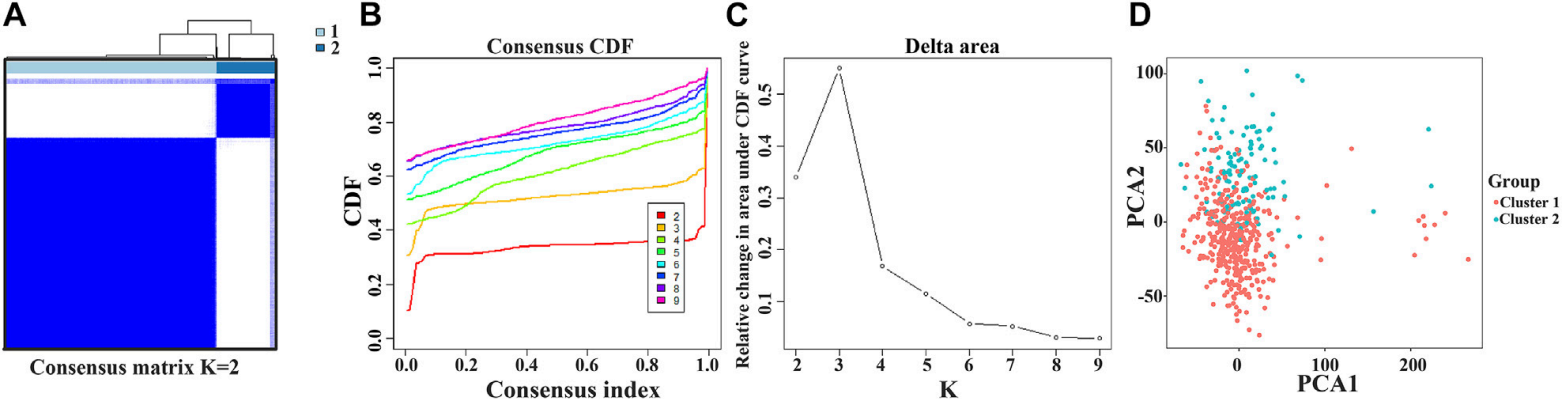
Consensus clustering of model factors in LUAD. Note: LUAD, lung adenocarcinoma.

The NEK2, TOP2A, PLK1, CA4, CDK5R1, AURKB, and F2 genes were analyzed *via* cluster analysis. As the clustering evolves from *k* = 2 to 9, *k* = 2 might be the most appropriate choice for minimal interference (Figures 8A–C). Therefore, *k* = 2 was used for consensus cluster analysis, defined as the cluster1 and cluster2 groups. PCA analysis was performed in 535 LUAD tissues from the TCGA database, and the results revealed that the cluster1 and cluster2 groups were significantly different (Figure 8D).

### Possible Mechanisms of Lung Adenocarcinoma Progression in the High-Risk Model

**FIGURE 9:**
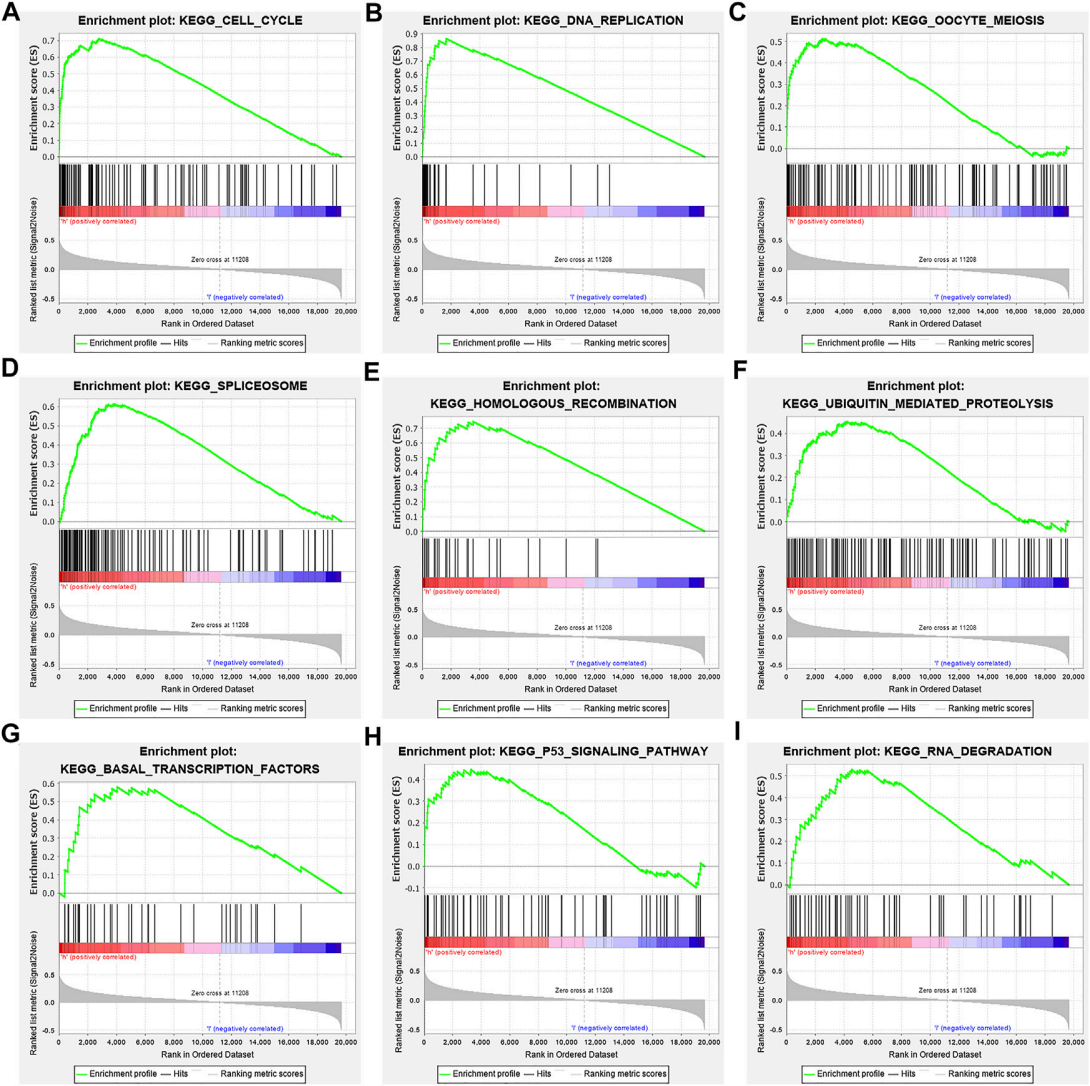
Signaling pathways involved in the risk model *via* the GSEA. Note: GSEA, gene set enrichment analysis.

In the high-risk group, the risk model involved the cell cycle, DNA replication, oocyte meiosis, spliceosome, homologous recombination, base excision repair, ubiquitin-mediated proteolysis, basal transcription factors, and other signaling pathways (Figure 9; Table 6).

**TABLE 6:** Signaling pathways involved in the risk model *via* the GSEA.

Signaling pathways	Size	ES	NES	NOM P
Cell cycle	124	0.70990354	2.4423828	0
DNA replication	36	0.8640126	2.1761284	0
Pyrimidine metabolism	98	0.5643264	2.162213	0
Mismatch repair	23	0.80003977	2.1095266	0
Oocyte meiosis	112	0.51308024	2.0650868	0
Spliceosome	126	0.61368227	2.0249872	0
Homologous recombination	28	0.74451005	2.0070393	0
Base excision repair	33	0.6601333	1.9589522	0.002
Nucleotide excision repair	44	0.5949838	1.9288769	0.002020202
Pentose phosphate pathway	27	0.61232996	1.9147793	0
Pathogenic *escherichia coli* infection	55	0.5294746	1.9063685	0.001976285
One carbon pool by folate	17	0.6632114	1.8398011	0.00407332
Purine metabolism	157	0.41026247	1.8340374	0
Progesterone mediated oocyte maturation	85	0.45555255	1.8318492	0.006160164
Ubiquitin mediated proteolysis	133	0.45231605	1.825182	0
Basal transcription factors	35	0.57929325	1.820776	0.002096436
N glycan biosynthesis	46	0.5264223	1.8005463	0.007905139
Glyoxylate and dicarboxylate metabolism	16	0.6469504	1.7673624	0.003898636
P53 signaling pathway	68	0.44462463	1.7558541	0.004201681
Proteasome	44	0.6673461	1.7501332	0.033797216
RNA degradation	57	0.5291112	1.7469671	0.008247423
Riboflavin metabolism	15	0.5477243	1.6133751	0.018036073
Glycolysis gluconeogenesis	62	0.43544966	1.5784576	0.015968064
Fructose and mannose metabolism	33	0.4526228	1.531549	0.040935673

ES, enrichment score; NES, normalized enrichment score; GSEA, gene set enrichment analysis.

## Discussion

Quercetin has been shown to exert a beneficial effect in cancer treatment, and could delay the progression of tumors (Zheng et al., 2012; Lee et al., 2015; Sonoki et al., 2015; Reyes-Farias and Carrasco-Pozo, 2019; Zhao et al., 2019; Li et al., 2020). For example, quercetin can inhibit the expression of Bcl-2 and promote the Bax expression to increase the apoptosis rate of A549 cells (Zheng et al., 2012). Claudin-2 is highly expressed in LUAD tissues and cells. Knockout of claudin-2 reduces the proliferation and migration of LUAD cells. Quercetin reduces the expression of claudin-2, and promotes the expression of miR-16 in LUAD A549 cells. However, miR-16 inhibitors can rescue the decreased expression of claudin-2 induced by quercetin (Sonoki et al., 2015). This demonstrates that quercetin has an anti-tumor progression effect in LUAD. At present, many studies have reported the mechanism of quercetin in the progress of LUAD. However, the roles of quercetin in the progress of LUAD has not yet been fully revealed. Therefore, this study uses biological methods to find quercetin target genes, and the risk model was built by screening valuable target genes to provide a new direction and vision for quercetin treatment in LUAD.

A growing body of studies has shown that regulating gene expression is a potentially effective method to delay the progress of LUAD (Sekimoto et al., 2017; Guo W et al., 2020; Hu et al., 2020; Kou et al., 2020; Huang et al., 2021). Hu et al. reported that miR-486-5p was downregulated in cancer patient tissues and serum exosomes, inhibiting the proliferation, and metastasis of LUAD cells. In contrast, up-regulating NEK2 expression eliminates the inhibitory effect of miR-486-5p overexpression on the progress of LUAD (Hu et al., 2020). TOP2A is highly expressed in LUAD tissues. TOP2A is an independent prognostic factor for LUAD patients (Guo W et al., 2020; Kou et al., 2020). Interfering with the expression of TOP2A in A549 and GLC82 cells has been reported to inhibit the proliferation, migration and invasion of LUAD cells and reduce the expression levels of CCNB1 and CCNB2 (Kou et al., 2020). In our study, ACHE, NEK2, TOP2A, CCNB2, TERT, PLK1, CA4, CDK1, PLA2G1B, CDK5R1, GPR35, AURKB, and F2 were independent factors affecting the prognosis of LUAD patients *via* the univariate Cox regression analysis. Furthermore, NEK2, TOP2A, PLK1, CA4, CDK5R1, AURKB, and F2 were independent factors affecting the prognosis of LUAD patients *via* the multivariate Cox regression analysis. K-M survival analysis demonstrated that the LUAD patients in the high-risk group had the poorer prognosis. Correlation analysis showed that the riskscore was related to the gender, clinical stage, T stage, lymph node metastasis, and survival status of LUAD patients. Consequently, univariate and multivariate Cox regression analysis revealed that the high riskscore was a risk factor for poor prognosis in LUAD patients. It has been reported in previous literature that NEK2, TOP2A, PLK1, CA4, and AURKB can play oncogenic or tumor suppressing roles in the progression of lung cancer (Bertran-Alamillo et al., 2019; Chen et al., 2020; Du et al., 2020; Gong et al., 2020; Shin et al., 2020). For example, TOP2A expression level was elevated in LUAD tissues and cells. TOP2A overexpression is associated with the prognosis in LUAD patients. The proliferation ability of A549 cells decreased and the apoptosis rate increased when TOP2A was inhibited in LUAD cells. Decreased TOP2A expression level could regulate LUAD growth by activating the ERK/JNK/p-P38/CHOP signaling pathway (Du et al., 2020). PLK1 expression level is the predictor of poor prognosis in patients with metastatic NSCLC. Active PLK1 promotes cancer metastasis by upregulating TGF-β signaling and amplifies metastatic properties by forming a positive feedback loop (Shin et al., 2020). It preliminarily showed that the quercetin target genes have crucial biological functions in LUAD progress.

Studies have found that NEK2, TOP2A, PLK1, CA4, CDK5R1, AURKB, and F2 have important regulatory roles in cell cycle, DNA replication, homologous recombination, and others (Chabalier-Taste et al., 2016; Fan et al., 2019; Zeng et al., 2019; Wang et al., 2020; Ha et al., 2022). The expression level of NEK2 in human gastric cancer (GC) tissues is significantly upregulated. The expression level of ERK in human tissues is correlated with the expression of NEK2, and their overexpression might predict a poor prognosis in GC patients. Inhibiting NEK2 expression in GC cells can attenuate the ERK and c-JUN phosphorylation, reducing the cyclin D1 transcription. NEK2 can rescue the GC cell viability, proliferation and cell cycle progression (Fan et al., 2019). TOP2A was overexpressed in bladder urothelial carcinoma (BLCA) samples compared with normal epithelial tissue. Univariate Cox regression analysis showed that high expression of TOP2A was a factor affecting cancer-specific survival, PFS and recurrence-free survival of BLCA patients. Inhibiting the expression of TOP2A in BLCA cells could inhibit cell proliferation, migration and invasion, and promote anti-apoptosis (Zeng et al., 2019). Furthermore, the results of GO and KEGG showed that the DEGs of quercetin are involved in cell proliferation, G2/M transition of the mitotic cell cycle, positive regulation of the reactive oxygen species metabolic process, mitotic nuclear division, oocyte meiosis, etc. GSEA results demonstrated that the risk model involved signaling pathways such as cell cycle, DNA replication, oocyte meiosis, spliceosome, homologous recombination, base excision repair, ubiquitin-mediated proteolysis, and basal transcription factors. However, further research is required to confirm those results in the future.

This bioinformatics results revealed the potential role of hub quercetin target genes in the progression of LUAD and constructed a risk model to provide potential therapeutic targets for quercetin treatment of LUAD patients. However, our research results remain to be confirmed by basic research in the future. The prognostic value of the cellular level model was evaluated to explore the impact of quercetin on hub targets, as well as the expression of hub target genes in LUAD. Our results preliminarily indicated that the quercetin target genes NEK2, TOP2A, PLK1, CA4, CDK5R1, AURKB, and F2 were abnormally expressed in LUAD tissues, and their expression levels were related to the diagnosis and prognosis of LUAD patients. The constructed risk model is expected to effectively evaluate the prognosis of LUAD patients. In conclusion, the quercetin potential target genes NEK2, TOP2A, PLK1, CA4, CDK5R1, AURKB, and F2, were abnormally expressed in LUAD tissues, and their expression levels were related to the diagnosis and prognosis of LUAD patients. The constructed risk model is expected to effectively evaluate the prognosis of LUAD patients.

## Data Availability

The original contributions presented in the study are included in the article/Supplementary Material, further inquiries can be directed to the corresponding authors.
