# Supplementary material for: A New Risk Model Based on 7 Quercetin-Related Target Genes for Predicting the Prognosis of Patients With Lung Adenocarcinoma
Source: Front Genet. 2022 May 13;13:890079. doi: 10.3389/fgene.2022.890079 (PMC9136292; doi:10.3389/fgene.2022.890079)
Supplement: Supplementary file 1 [file DataSheet1.docx]

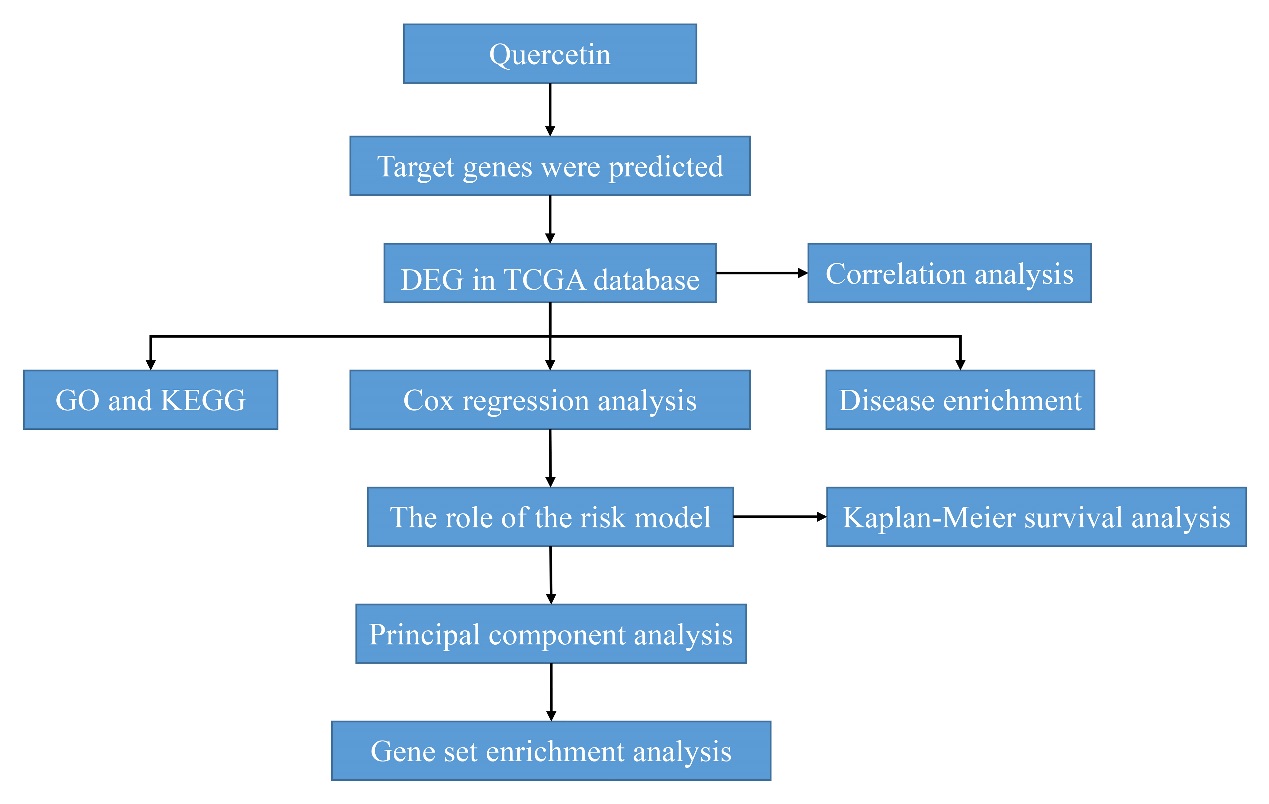
Figure S1. Technology roadmap under study.


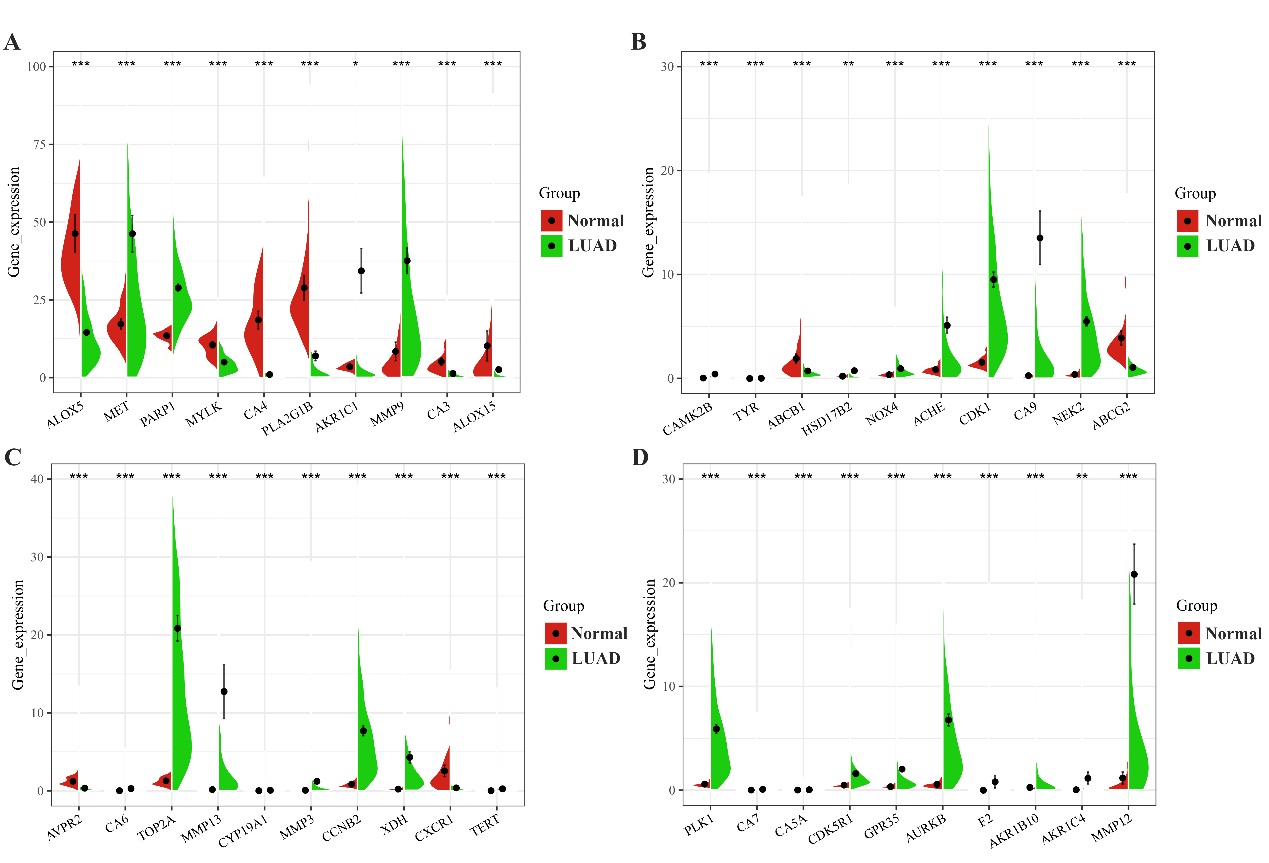
Figure S2. 40 DEGs with a fold change greater than 1 in LUAD tissues.

Note: DEGs, differentially expressed genes; LUAD, lung adenocarcinoma.


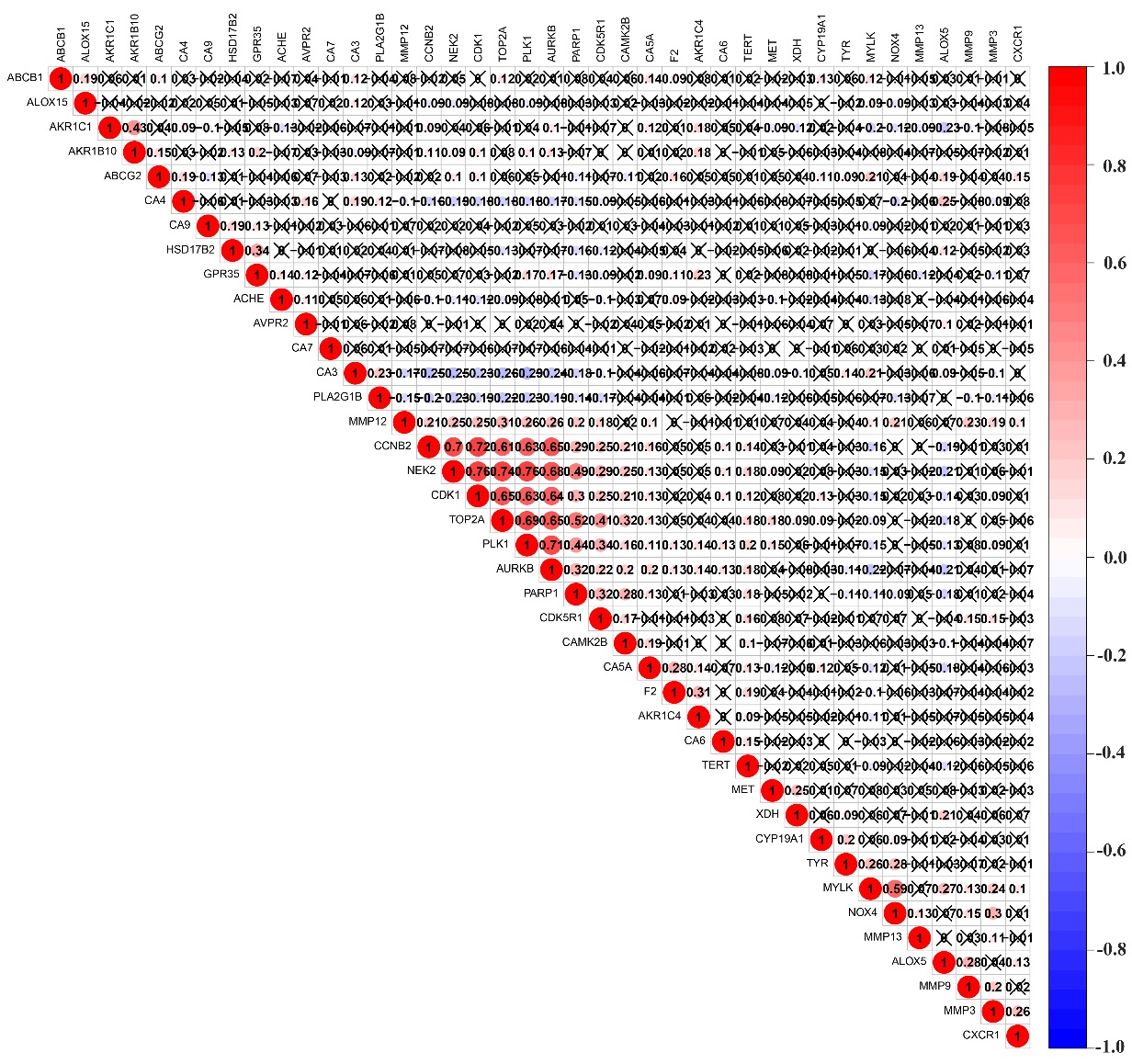
Figure S3. The correlation between the differential gene expression levels in 535 LUAD tissues.

Note: LUAD, lung adenocarcinoma.


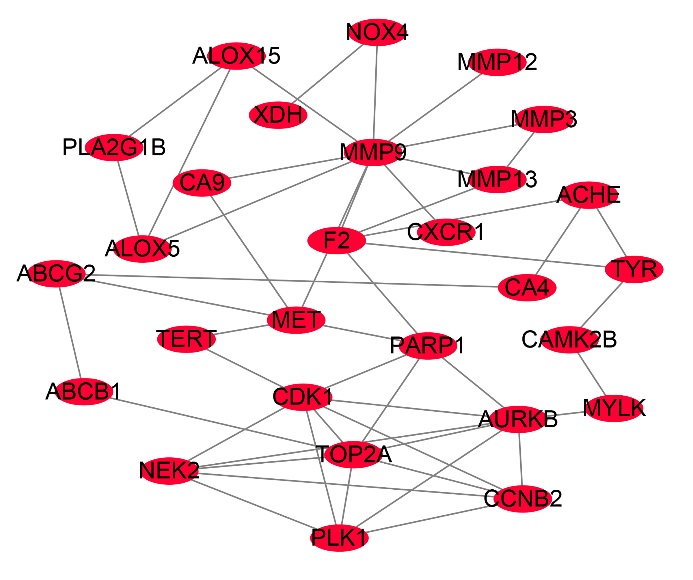


Figure S4. The PPI network of the DEGs was constructed in the String database.

Note: DEGs, differentially expressed genes; PPI, Protein-protein interaction.


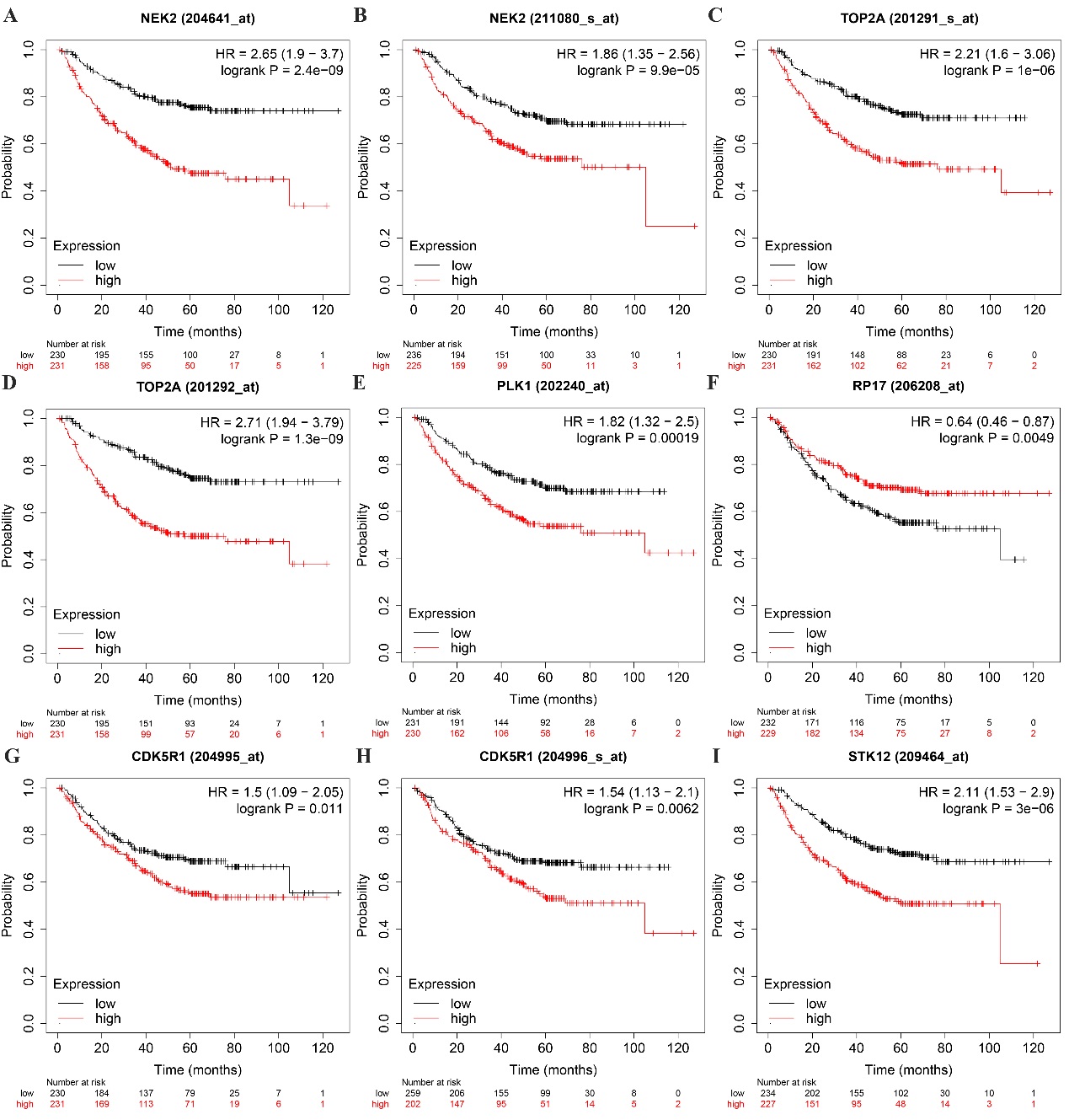
Figure S5. The expression levels of model factors were correlated with the progression-free survival in LUAD patients in the Kaplan-Meier Plotter database.

Table S1. The quercetin target genes in the SwissTargetPrediction database.

| Gene | Description | Classification |
| --- | --- | --- |
| MAPT | Microtubule-associated protein tau | Unclassified protein |
| AHR | Aryl hydrocarbon receptor | Transcription factor |
| TTR | Transthyretin | Secreted protein |
| F2 | Thrombin | Protease |
| MMP13 | Matrix metalloproteinase 13 | Protease |
| MMP3 | Matrix metalloproteinase 3 | Protease |
| MMP9 | Matrix metalloproteinase 9 | Protease |
| MMP2 | Matrix metalloproteinase 2 | Protease |
| BACE1 | Beta-secretase 1 | Protease |
| MMP12 | Matrix metalloproteinase 12 | Protease |
| ABCC1 | Multidrug resistance-associated protein 1 | Primary active transporter |
| ABCB1 | P-glycoprotein 1 | Primary active transporter |
| ABCG2 | ATP-binding cassette sub-family G member 2 | Primary active transporter |
| PTPRS | Receptor-type tyrosine-protein phosphatase S | Phosphatase |
| XDH | Xanthine dehydrogenase | Oxidoreductase |
| MAOA | Monoamine oxidase A | Oxidoreductase |
| ALOX5 | Arachidonate 5-lipoxygenase | Oxidoreductase |
| TYR | Tyrosinase | Oxidoreductase |
| CCNB3 | Cyclin B3 | Other cytosolic protein |
| CDK1 | Cyclin-dependent kinase 1 | Other cytosolic protein |
| CCNB1 | Cyclin B1 | Other cytosolic protein |
| CCNB2 | Cyclin B2 | Other cytosolic protein |
| ESR2 | Estrogen receptor beta | Nuclear receptor |
| ESRRA | Estrogen-related receptor alpha | Nuclear receptor |
| APP | Beta amyloid A4 protein | Membrane receptor |
| CA2 | Carbonic anhydrase II | Lyase |
| CA7 | Carbonic anhydrase VII | Lyase |
| CA1 | Carbonic anhydrase I | Lyase |
| CA3 | Carbonic anhydrase III | Lyase |
| CA6 | Carbonic anhydrase VI | Lyase |
| CA12 | Carbonic anhydrase XII | Lyase |
| CA14 | Carbonic anhydrase XIV | Lyase |
| CA9 | Carbonic anhydrase IX | Lyase |
| CA4 | Carbonic anhydrase IV | Lyase |
| CA5A | Carbonic anhydrase VA | Lyase |
| CA13 | Carbonic anhydrase XIII | Lyase |
| IGF1R | Insulin-like growth factor I receptor | Kinase |
| FLT3 | Tyrosine-protein kinase receptor FLT3 | Kinase |
| EGFR | Epidermal growth factor receptor erbB1 | Kinase |
| PIM1 | Serine/threonine-protein kinase PIM1 | Kinase |
| AURKB | Serine/threonine-protein kinase Aurora-B | Kinase |
| DAPK1 | Death-associated protein kinase 1 | Kinase |
| GSK3B | Glycogen synthase kinase-3 beta | Kinase |
| SRC | Tyrosine-protein kinase SRC | Kinase |
| PTK2 | Focal adhesion kinase 1 | Kinase |
| KDR | Vascular endothelial growth factor receptor 2 | Kinase |
| PLK1 | Serine/threonine-protein kinase PLK1 | Kinase |
| CDK1 | Cyclin-dependent kinase 1 | Kinase |
| PKN1 | Protein kinase N1 | Kinase |
| CSNK2A1 | Casein kinase II alpha | Kinase |
| MET | Hepatocyte growth factor receptor | Kinase |
| NEK2 | Serine/threonine-protein kinase NEK2 | Kinase |
| CAMK2B | CaM kinase II beta | Kinase |
| ALK | ALK tyrosine kinase receptor | Kinase |
| AKT1 | Serine/threonine-protein kinase AKT | Kinase |
| NEK6 | Serine/threonine-protein kinase NEK6 | Kinase |
| AXL | Tyrosine-protein kinase receptor UFO | Kinase |
| NUAK1 | NUAK family SNF1-like kinase 1 | Kinase |
| SYK | Tyrosine-protein kinase SYK | Kinase |
| INSR | Insulin receptor | Kinase |
| MYLK | Myosin light chain kinase, smooth muscle | Kinase |
| CDK5 | Cyclin-dependent kinase 5 | Kinase |
| CDK5R1 | CDK5 activator 1 | Kinase |
| CDK6 | Cyclin-dependent kinase 6 | Kinase |
| CDK2 | Cyclin-dependent kinase 2 | Kinase |
| TOP2A | DNA topoisomerase II alpha | Isomerase |
| TOP1 | DNA topoisomerase I | Isomerase |
| ACHE | Acetylcholinesterase | Hydrolase |
| AVPR2 | Vasopressin V2 receptor | Family A G protein-coupled receptor |
| DRD4 | Dopamine D4 receptor | Family A G protein-coupled receptor |
| ADORA1 | Adenosine A1 receptor | Family A G protein-coupled receptor |
| ADORA2A | Adenosine A2a receptor | Family A G protein-coupled receptor |
| CXCR1 | Interleukin-8 receptor A | Family A G protein-coupled receptor |
| GPR35 | G-protein coupled receptor 35 | Family A G protein-coupled receptor |
| KDM4E | Lysine-specific demethylase 4D-like | Eraser |
| NOX4 | NADPH oxidase 4 | Enzyme |
| AKR1B1 | Aldose reductase | Enzyme |
| GLO1 | Glyoxalase I | Enzyme |
| MPO | Myeloperoxidase | Enzyme |
| PIK3R1 | PI3-kinase p85-alpha subunit | Enzyme |
| PYGL | Liver glycogen phosphorylase | Enzyme |
| HSD17B2 | Estradiol 17-beta-dehydrogenase 2 | Enzyme |
| ALOX15 | Arachidonate 15-lipoxygenase | Enzyme |
| ALOX12 | Arachidonate 12-lipoxygenase | Enzyme |
| PLA2G1B | Phospholipase A2 group 1B | Enzyme |
| AKR1C2 | Aldo-keto reductase family 1 member C2 | Enzyme |
| AKR1C1 | Aldo-keto reductase family 1 member C1 | Enzyme |
| AKR1C3 | Aldo-keto-reductase family 1 member C3 | Enzyme |
| AKR1C4 | Aldo-keto reductase family 1 member C4 | Enzyme |
| AKR1A1 | Aldehyde reductase (by homology) | Enzyme |
| PIK3CG | PI3-kinase p110-gamma subunit | Enzyme |
| APEX1 | DNA-(apurinic or apyrimidinic site) lyase | Enzyme |
| ARG1 | Arginase-1 | Enzyme |
| MPG | DNA-3-methyladenine glycosylase | Enzyme |
| HSD17B1 | Estradiol 17-beta-dehydrogenase 1 | Enzyme |
| PARP1 | Poly [ADP-ribose] polymerase-1 | Enzyme |
| CD38 | Lymphocyte differentiation antigen CD38 | Enzyme |
| AKR1B10 | Aldo-keto reductase family 1 member B10 | Enzyme |
| TNKS2 | Tankyrase-2 | Enzyme |
| TNKS | Tankyrase-1 | Enzyme |
| TERT | Telomerase reverse transcriptase | Enzyme |
| SLC22A12 | Solute carrier family 22 member 12 | Electrochemical transporter |
| CYP19A1 | Cytochrome P450 19A1 | Cytochrome P450 |
| CYP1B1 | Cytochrome P450 1B1 | Cytochrome P450 |

Table S2. Differentially expressed genes in LUAD tissues.

| Gene | Normal | LUAD | logFC | P value |
| --- | --- | --- | --- | --- |
| CD38 | 4.236592259 | 3.815805619 | -0.150916606 | 0.015778722 |
| MPO | 0.221803434 | 0.166627919 | -0.412651555 | 2.82E-07 |
| ALOX5 | 46.25523982 | 14.52732521 | -1.670847708 | 5.93E-29 |
| CAMK2B | 0.049236775 | 0.421586942 | 3.098022011 | 0.000194154 |
| MYLK | 10.57165139 | 5.023807105 | -1.073347781 | 8.14E-21 |
| DRD4 | 0.294873176 | 0.573480796 | 0.959650587 | 0.002651423 |
| NUAK1 | 5.041261385 | 4.210123518 | -0.259922199 | 0.005764131 |
| TYR | 0.000583999 | 0.00931391 | 3.995349033 | 8.77E-05 |
| GSK3B | 9.216705514 | 8.436194015 | -0.127658881 | 0.000372776 |
| ABCB1 | 1.908949012 | 0.719293508 | -1.40812608 | 2.14E-14 |
| AKR1B1 | 21.2622954 | 27.73137768 | 0.383221937 | 0.039028627 |
| HSD17B2 | 0.222337618 | 0.7371236 | 1.729154488 | 0.001096623 |
| NOX4 | 0.344120878 | 0.952886641 | 1.469389171 | 2.16E-12 |
| ACHE | 0.852870206 | 5.110213705 | 2.582985518 | 3.06E-08 |
| PYGL | 8.986823204 | 15.18873699 | 0.757118783 | 0.000202312 |
| APEX1 | 44.09076185 | 81.30129575 | 0.88280194 | 1.61E-25 |
| MMP9 | 8.445666361 | 37.58516834 | 2.153880305 | 7.65E-16 |
| CSNK2A1 | 11.22268101 | 16.28650288 | 0.537259488 | 3.29E-16 |
| MPG | 12.857382 | 20.74573878 | 0.690218122 | 2.54E-14 |
| CA2 | 32.22621807 | 16.77188975 | -0.942189638 | 2.74E-25 |
| PTPRS | 6.626407017 | 5.681932814 | -0.221845049 | 0.000325873 |
| CDK6 | 2.901428473 | 3.185557368 | 0.134782455 | 0.000256819 |
| PIK3CG | 2.197853932 | 1.724176736 | -0.350187844 | 2.24E-07 |
| MET | 17.25153837 | 46.31649801 | 1.424801159 | 0.000129599 |
| CA9 | 0.269006771 | 13.52854628 | 5.652220522 | 1.57E-25 |
| HSD17B1 | 0.310385447 | 0.541914953 | 0.804005542 | 1.51E-07 |
| AKR1A1 | 30.95065342 | 44.2544804 | 0.515853656 | 1.18E-12 |
| NEK2 | 0.372781618 | 5.481985943 | 3.878296003 | 1.13E-33 |
| ARG1 | 0.292428532 | 0.15720036 | -0.895479556 | 9.93E-16 |
| ABCG2 | 3.891805777 | 1.047588969 | -1.89336694 | 5.70E-30 |
| NEK6 | 10.74668107 | 20.00392984 | 0.896392274 | 4.49E-22 |
| FLT3 | 0.376008289 | 0.341264179 | -0.139875477 | 0.00299809 |
| PKN1 | 34.65373274 | 30.95279915 | -0.162940879 | 0.000444277 |
| CDK2 | 6.078555701 | 9.339076855 | 0.619551378 | 1.50E-10 |
| GLO1 | 56.86376024 | 69.91509084 | 0.298094385 | 0.011592818 |
| AVPR2 | 1.234203422 | 0.358546929 | -1.783346333 | 1.39E-27 |
| KDR | 17.59518074 | 12.24652222 | -0.522808224 | 1.90E-10 |
| ADORA2A | 0.124295924 | 0.102372154 | -0.279955645 | 0.000186307 |
| CA6 | 0.011849034 | 0.281894217 | 4.572312488 | 0.000492085 |
| TOP2A | 1.273108254 | 20.86271818 | 4.034500134 | 4.34E-35 |
| CA1 | 0.192479411 | 0.169989676 | -0.179257002 | 2.20E-19 |
| MMP13 | 0.172913296 | 12.74525631 | 6.203767768 | 4.26E-26 |
| CYP19A1 | 0.033011161 | 0.096082436 | 1.541318843 | 6.80E-08 |
| PARP1 | 13.51054369 | 28.90555188 | 1.097260884 | 5.56E-28 |
| PIK3R1 | 9.250435134 | 5.086449775 | -0.862862191 | 2.62E-21 |
| CCNB3 | 0.186741149 | 0.13567659 | -0.460868048 | 1.52E-09 |
| MMP3 | 0.074374208 | 1.225321 | 4.042213541 | 3.35E-18 |
| CCNB2 | 0.85272533 | 7.724280418 | 3.179247523 | 3.47E-33 |
| XDH | 0.236332784 | 4.310917397 | 4.18910324 | 3.48E-28 |
| ALOX15 | 10.25568279 | 2.588697597 | -1.986125195 | 1.07E-12 |
| CXCR1 | 2.556918658 | 0.390156367 | -2.712281917 | 1.80E-23 |
| TERT | 0.00486791 | 0.25059112 | 5.68588908 | 4.03E-31 |
| CA3 | 5.164310546 | 1.361228429 | -1.92366657 | 2.46E-22 |
| CDK5 | 3.476719265 | 6.347503646 | 0.86846274 | 1.43E-20 |
| SYK | 15.06620655 | 13.37571597 | -0.171700095 | 0.003518611 |
| PLK1 | 0.577680656 | 5.911287315 | 3.355128254 | 2.65E-34 |
| CA4 | 18.53023779 | 1.012544663 | -4.193823944 | 3.12E-34 |
| AXL | 21.7225352 | 12.05171449 | -0.849954087 | 2.15E-20 |
| CA7 | 0.011111517 | 0.084740592 | 2.930997398 | 4.25E-13 |
| PTK2 | 7.490854369 | 11.22659957 | 0.583718835 | 3.98E-11 |
| CDK1 | 1.559918253 | 9.505668682 | 2.60731769 | 3.27E-29 |
| PLA2G1B | 28.89106922 | 6.982282146 | -2.048853037 | 2.94E-28 |
| INSR | 6.33318106 | 8.276431159 | 0.386078479 | 0.00444343 |
| ESRRA | 11.85823881 | 17.05856884 | 0.524606858 | 2.56E-10 |
| CA5A | 0.008305728 | 0.038347433 | 2.206951378 | 1.22E-10 |
| CDK5R1 | 0.49372022 | 1.592183329 | 1.689240827 | 6.69E-21 |
| GPR35 | 0.34123397 | 2.031166595 | 2.573475395 | 6.85E-15 |
| AURKB | 0.562161788 | 6.770060245 | 3.590111376 | 8.17E-33 |
| F2 | 0.004497974 | 0.803636905 | 7.481124576 | 3.09E-09 |
| BACE1 | 7.20916781 | 5.286150668 | -0.447615186 | 2.68E-12 |
| MAPT | 0.399909758 | 0.324455597 | -0.301653431 | 5.46E-08 |
| AKR1C1 | 3.523312266 | 34.33050626 | 3.284486883 | 0.018253344 |
| MAOA | 43.29204931 | 26.74996077 | -0.694565319 | 1.90E-16 |
| DAPK1 | 21.13842051 | 11.02016395 | -0.939721893 | 3.05E-20 |
| SRC | 9.428636466 | 15.12407092 | 0.681725466 | 4.50E-13 |
| AKR1B10 | 0.273728493 | 50.59486695 | 7.530101598 | 3.24E-14 |
| AKR1C4 | 0.0328767 | 1.159276664 | 5.140015589 | 0.006154793 |
| TOP1 | 34.6837334 | 48.89466697 | 0.495417916 | 7.97E-12 |
| MMP12 | 1.174265463 | 20.82348939 | 4.148381345 | 4.56E-23 |

Note: LUAD, lung adenocarcinoma.
